# Radiation Increases Functional KCa3.1 Expression and Invasiveness in Glioblastoma

**DOI:** 10.3390/cancers11030279

**Published:** 2019-02-26

**Authors:** Giuseppina D’Alessandro, Lucia Monaco, Luigi Catacuzzeno, Fabrizio Antonangeli, Antonio Santoro, Vincenzo Esposito, Fabio Franciolini, Heike Wulff, Cristina Limatola

**Affiliations:** 1 Department of Physiology and Pharmacology, Sapienza University of Rome, 00185 Rome, Italy; giuseppina.dalessandro@uniroma1.it (G.D.); lucia.monaco@uniroma1.it (L.M.); 2 IRCCS Neuromed, Via Atinense, 86077 Pozzilli, Italy; vincenzo.esposito@uniroma1.it; 3 Department of Chemistry Biology and Biotechnology, University of Perugia, 06123 Perugia, Italy; luigi.catacuzzeno@unipg.it (L.C.); Fabio.franciolini@unipg.it (F.F.); 4 Department of Molecular Medicine, Sapienza University, Laboratory associated to Istituto Pasteur Italia—Fondazione Cenci Bolognetti, 00161 Rome, Italy; fabrizio.antonangeli@uniroma1.it; 5 Department of Neurology and Psychiatry, Sapienza University, 00185 Rome, Italy; antonio.santoro@uniroma1.it; 6 Department of Pharmacology, University of California, Davis, CA 95616, USA; hwulff@ucdavis.edu; 7 Department of Physiology and Pharmacology Sapienza University of Rome, Laboratory associated to Istituto Pasteur Italia, 00185 Rome, Italy

**Keywords:** Glioblastoma, KCa3.1, radiation, invasion, IL-4

## Abstract

Glioblastoma (GBM) is a deadly brain tumor, with fast recurrence even after surgical intervention, radio- and chemotherapies. One of the reasons for relapse is the early invasion of surrounding brain parenchyma by GBM, rendering tumor eradication difficult. Recent studies demonstrate that, in addition to eliminate possible residual tumoral cells after surgery, radiation stimulates the infiltrative behavior of GBM cells. The intermediate conductance of Ca^2+^-activated potassium channels (KCa3.1) play an important role in regulating the migration of GBM. Here, we show that high dose radiation of patient-derived GBM cells increases their invasion, and induces the transcription of key genes related to these functions, including the *IL-4/IL-4R* pair. In addition, we demonstrate that radiation increases the expression of KCa3.1 channels, and that their pharmacological inhibition counteracts the pro-invasive phenotype induced by radiation in tumor cells. Our data describe a possible approach to treat tumor resistance that follows radiation therapy in GBM patients.

## 1. Introduction

Glioblastoma (GBM) is the most common and deadly primary brain tumor in adults [1]. Despite the current standard multimodal treatment regimen, including surgery, and/or adjuvant chemo- and radiotherapy (RT), mean survival in GBM patients remains low (less than 15 months) [2,3]. This poor patient prognosis results from the high proliferation rate of GBM cells, and from the ability of these cells to spread into surrounding brain parenchyma, thus escaping surgical resection, with rapid appearance of new tumor masses. It has been shown that 80% of tumor recurrence occurs at the resection margins, where the highest radiation doses are delivered [4]. However, invading GBM cells intrinsically show decreased susceptibility to apoptosis, making radiotherapy and chemotherapy, as well as radiation surgery, poorly effective [5,6]. For all these reasons, new strategies to suppress GBM cell migration are urgently needed. Recent studies demonstrated that radiation induces GBM cell migration, acting both on tumor cells, increasing invasiveness index, and on normal brain parenchyma, increasing chemokine secretion (such as CXCL12) from stromal cells [7,8,9]. Autocrine and paracrine CXCL12 is a signal which determines the specific invasion pattern of GBM cells in the brain, the so-called Sherer’s structures [10]. However, cell invasion also requires the activity of various calcium and volume-sensitive ion channels located at the front and the rear edges of migrating cells [11]. Among the ion channels activated during GBM cell migration, growing attention is given to potassium channels, in particular those belonging to the family of calcium-activated potassium channels [12,13]. Previous studies demonstrated that the inhibition of the intermediate-conductance calcium activated potassium channel KCa3.1 blocked CXCL12-induced migration in human primary GBM cells [14,15]. Our current study shows that treatment with a single high radiation, even higher than that administered in radiosurgery for recurrent GBM [16], increases the migration and invasion of the human GL-15 cell line and of some primary patient-derived human GBM cells. Radiation also induces the transcription of key genes related to migration and invasion, such as CXCL12 and CXCR4, metalloproteinases, and some transcription factors. In addition, we describe that radiation induces the functional over-expression of KCa3.1 channels, and that the inhibition of these channels with 1-[(2-Chlorophenyl)diphenylmethyl]-1H-pyrazole (TRAM-34) counteracts the expression of the pro-invasive genes induced by radiation in tumor cells. Among the genes analyzed, we measured the level of interleukin-4 (IL-4), a T helper type 2 (TH2) cytokine, and its receptor (IL-4R). IL-4 suppresses the cancer-directed immune surveillance of myeloid cells and increases tumor metastasis in carcinoma [17]. We described, for the first time, that high radiation treatment increases *IL-4* and *IL-4R* mRNA as well as IL-4 protein in irradiated GBM cells. Considering that AP-1 controls the transcription of the KCa3.1 gene *KCNN4* [18] and that IL-4/IL-4R signaling regulates KCa3.1 expression through the activation of the AP-1 transcription factor [19], this signaling could be relevant upon GBM radiation. Our results suggest a possible new approach to counteract radiation-induced GBM migration that follows radiosurgery in patients with recurrent GBM. Co-treatment with a KCa3.1 inhibitor and the currently approved drugs (e.g., Temozolomide) during the radiation protocol could decrease the induction of pro-invasive genes. Of note, the selective KCa3.1 inhibitor used in this work (TRAM-34) has a structural analogue drug, Senicapoc^®^, which has been already used in clinical trials for sickle cell anemia and has been shown safe for patients [20].

## 2. Results

### 2.1. The Functional Expression of KCa3.1 Channels Increases in Irradiated Glioblastoma (GBM) Cells

**Figure 1:**
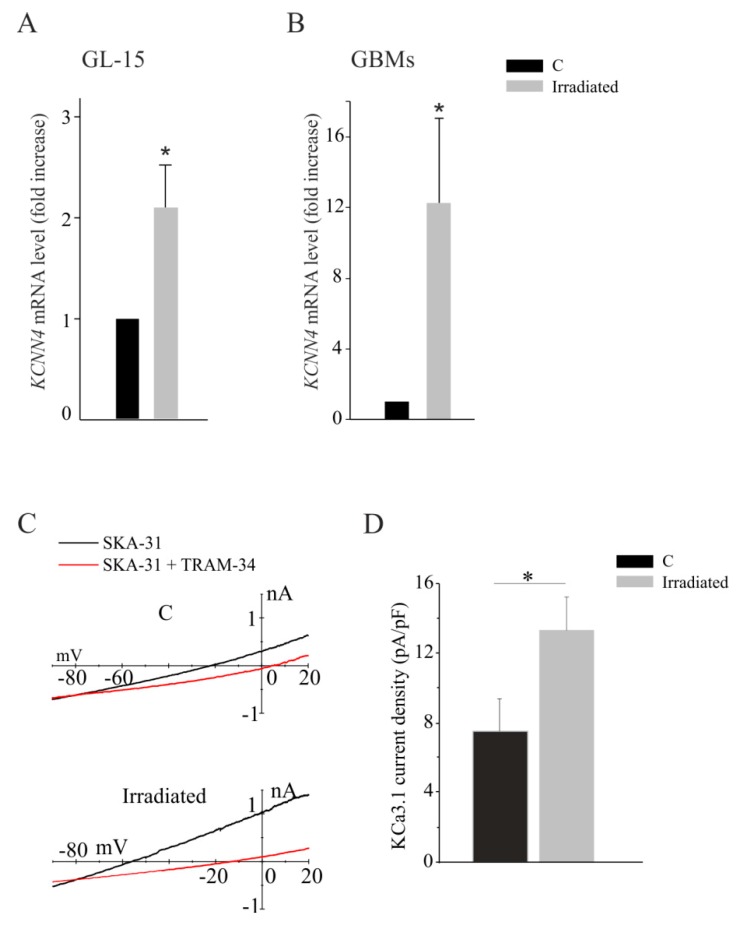
(**A**,**B**) Expression analysis by qRT-PCR of *KCNN4* mRNA in GL-15 cells (**A**), in patient-derived primary glioblastomas (GBMs) (GBM18, GBM19, and GBM45) (**B**) after 1 cycle of radiation (35 Gy). (**A**) * *p* < 0.05 vs. control (C) GL-15; (**B**) * *p* < 0.05 vs. respective controls (C), *n* = 3 (in duplicate); (**C**) Current traces obtained from control (C) and irradiated GL-15 cells applying 1 s long voltage ramps from −90 to +20 mV, from a holding potential of −60 mV. Data are shown as current–voltage relationships, by plotting the current amplitude as a function of the applied voltage. Black and red traces are in the presence of external SKA-31 (3 µM), and external SKA-31 (3 µM) + 1-[(2-Chlorophenyl)diphenylmethyl]-1H-pyrazole (TRAM)-34 (3 µM), respectively. (**D**) Mean KCa3.1 current density (current amplitude to electrical capacitance ratio) assessed at 0 mV as the TRAM-34 sensitive current. *N* = 11 cells for C and *n* = 16 cells for irradiated, * *p* < 0.05 vs. C.

We exposed a human GBM cell line (GL-15) and primary GBM cells derived from patients (GBM18, GBM19, and GBM45) to a single high radiation dose, higher than that usually administered to patients with recurrent GBM, in stereo radio-surgery [16]. For this reason, we first verified the survival of GL-15 cells, 72 h after irradiation protocol, by MTT assay. As shown in Supplementary Figure S1, the viability of irradiated GL-15 cells was similar to controls. To investigate the effect of radiation on KCa3.1 channel expression, human GBM cells were irradiated and analyzed by the qRT-PCR for *KCNN4* expression, after 72 h. As shown in Figure 1A, upon radiation, GL15 cells increased the expression of the *KCNN4* gene approximately two-fold. Similar results were obtained in primary GBM cells, where radiation increased the *KCNN4* level in each cell population, compared to their control (Figure 1B). We also evaluated the functional activity of KCa3.1 channels in GBM cells, by electrophysiological recordings, 48–72 h after irradiation. Figure 1C shows representative KCa3.1 current traces obtained in control or irradiated GL-15 cells. As shown in Figure 1D, and, in line with the mRNA expression, an increased potassium current with the pharmacological properties of KCa3.1 was observed in irradiated GL-15 cells.

### 2.2. KCa3.1 Inhibition Decreases Radiation-Induced Cell Migration and Invasion

**Figure 2:**
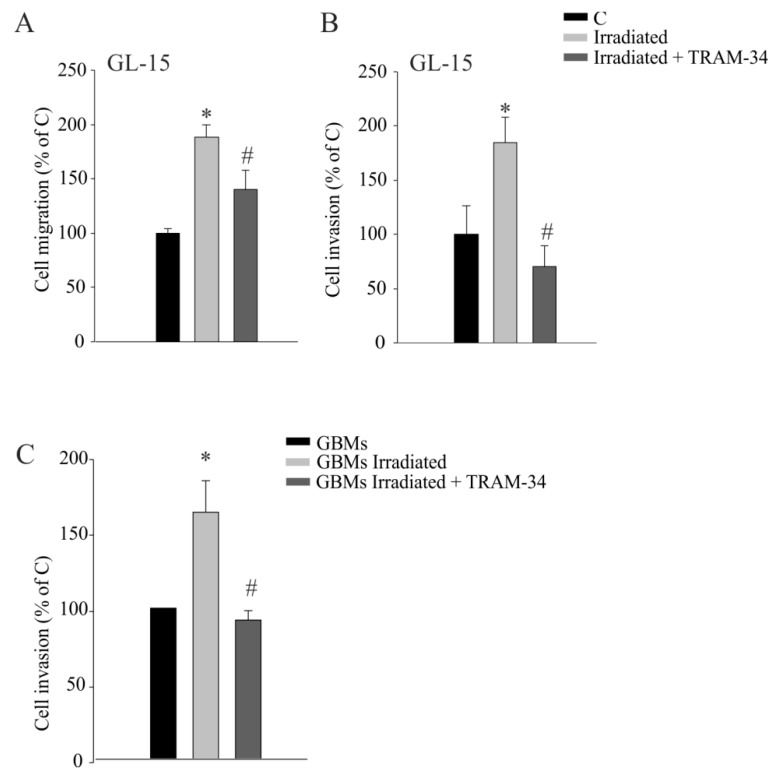
(**A**,**B**) Migration and invasion assays of irradiated GL-15 cells (after 24 h), in the presence or absence of 5 µM of TRAM-34. * *p* < 0.05 vs. control (C) GL-15. # *p* < 0.05 vs. irradiated GL-15 *n* = 3 (in duplicate); (**C**) Cell invasion assay of irradiated human primary GBMs (GBM18, GBM19, and GBM45) (after 24 h) in the presence or absence of TRAM-34 (5 µM for 24 h), * *p* < 0.05 vs. control GBMs (C). # *p* < 0.05 vs. irradiated GBMs. *n* = 3 (in duplicate).

We have previously shown that GBM cell migration and invasion, both in in vitro and in vivo experimental systems, can be induced and sustained by KCa3.1 activity [14,21]. To investigate whether the increased expression of KCa3.1 channels in irradiated GBM was associated with enhanced migration and invasion capabilities, these activities were tested in GL-15 cells in the presence of the KCa3.1 inhibitor, TRAM-34 (5 µM), 24 h after irradiation. As shown in Figure 2A,B, radiation induced an increase of basal migration and invasion through a layer of extracellular matrix (Matrigel). The inhibition of channel function significantly reduced the effects of radiation (Figure 2B). Invasion assay was also performed on human GBM18, GBM19, and GBM45 cells and, similarly, the increase of cell invasion induced by radiation was abolished by TRAM-34 (Figure 2C).

### 2.3. KCa3.1 Activity Is Involved in Radiation-Induced Transcription of Genes Related to GBM Cell Invasion

**Figure 3:**
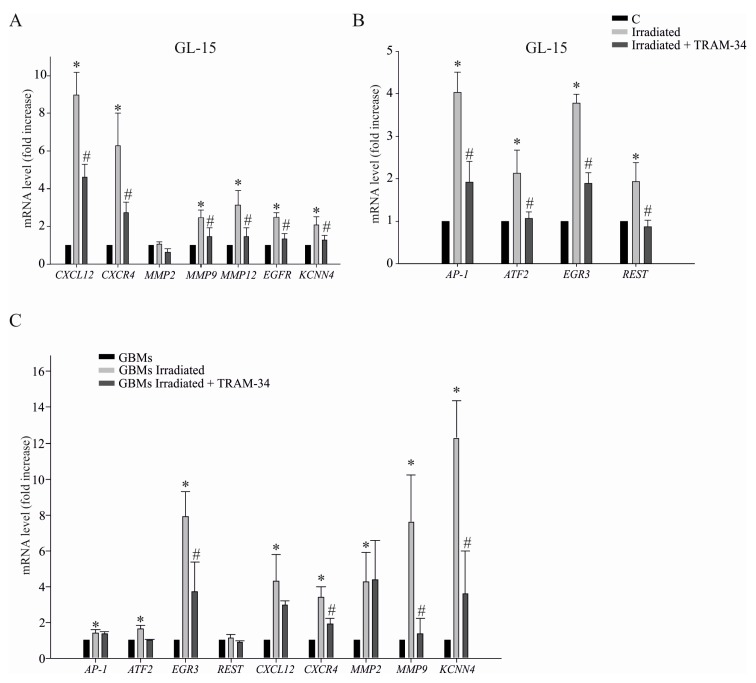
(**A**,**B**) Expression analysis by qRT-PCR of invasion-related genes in irradiated or control, (C) GL-15 cells upon TRAM-34 treatment (5 µM, 48 h); * *p* < 0.05 vs. control GL-15. # *p* < 0.05 vs. irradiated GL-15 *n* = 3 (in duplicate). (**C**) Same as in A and B for human primary GBM cells, upon TRAM-34 treatment (5 µM, 48 h); * *p* < 0.05 vs. GBMs. # *p* < 0.05 vs. irradiated GBMs *n* = 3 (in duplicate).

In order to reveal the mechanisms behind the increased invasive ability of irradiated GBM cells, we investigated the effects of irradiation on the mRNA expression of some key genes involved in cell invasion. We found that radiation induced the up-regulation of the chemokine CXCL12 and its receptor CXCR4, of the metalloproteinases 9 and 12 (MMP9 and MMP12), and of key transcription factors involved in the expression of genes related to migration of cancer cells. Among them, AP-1 (activating protein 1), ATF2 (activating transcription factor 2), EGR3 (early growth response 3), and REST (RE-1 Silencing transcription factor) (Figure 3A,B). In order to understand if KCa3.1 channel activity could contribute to the gene expression profile induced by radiation in GL-15 cells, cells were treated with TRAM-34 (48 h, 5 µM) and analyzed by qRT-PCR. Data shown in Figure 3A,B demonstrated that inhibition of KCa3.1 activity reduced the expression of genes induced by radiation. We also found that the expression of *KCNN4* (Figure 3A) and the corresponding KCa3.1 current was modulated by channel activity (Supplementary Figure S2). As shown in Figure 3C, similar results were obtained in primary GBM cells derived from patients, where radiation up-regulated 8 out of 9 tested genes, partially reduced by TRAM-34 treatment. The effects of radiation on gene expression in each cell population is shown in Supplementary Table S1. These data are in accordance with the increased ability of GBM cells to invade the extracellular matrix shown before, and describe an important effect of radiation as modulator of gene expression, increasing the tumor cell invasiveness.

### 2.4. Radiation-Induced IL-4/IL-4R Expression is Dependent on KCa3.1 Channel Activity

**Figure 4:**
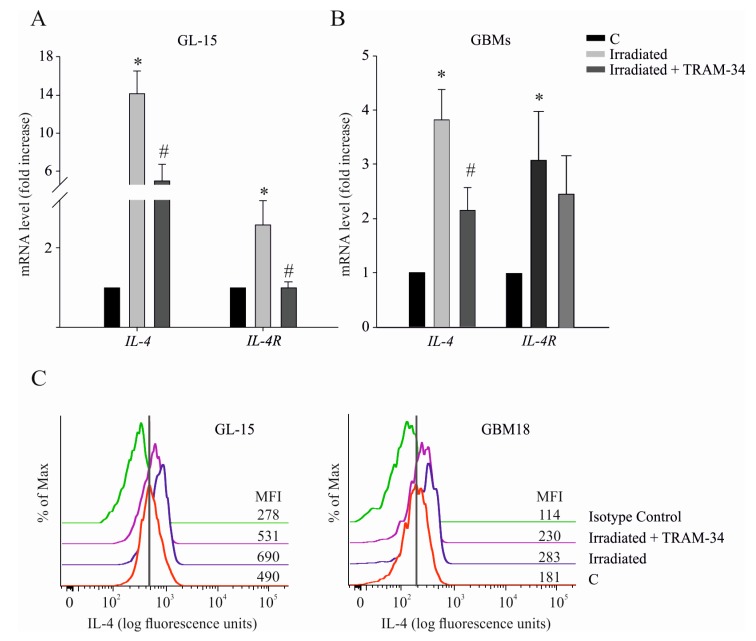
(**A**) Expression analysis by qRT-PCR of *IL-4* and *IL-4R* in irradiated or untreated (C) GL-15 cells after TRAM-34 treatment (5 µM, 48 h). * *p* < 0.05 vs. control GL-15. # *p* < 0.05 vs. irradiated GL-15 *n* = 3 (in duplicate). (**B**) Expression analysis, by qRT-PCR, of *IL-4* and *IL-4R* in GBMs (GBM18, GBM19, GBM45, and GBM137) * *p* < 0.05 vs. C; # *p* < 0.05 vs. irradiated GBM *n* = 4 (in duplicate). (**C**) IL-4 production by GL-15 and GBM18 cells, evaluated by intracellular immunostaining and flow cytometry analysis. Histograms are representative of two independent experiments. Isotype staining was performed on both control and irradiated cells. Gray line marks control histogram peak. The values shown represent the median fluorescence intensity (MFI) of each histogram/sample.

*KCNN4* transcription involves the IL-4/IL-4R axis in cultured rat and mouse microglia cells [18,22], and *IL-4R* and *KCNN4* expression in GBM negatively correlates with patient survival, as shown in the TCGA database (Supplementary Figure S3). In order to determine the effect of radiation on IL-4/IL-4R in GBM cells, we studied their expression in GL-15 cells and in human primary GBM cells. As shown in Figure 4A, *IL-4* and *IL-4R* transcription significantly increased upon irradiation in GL-15 cells, compared to controls. Inhibition of KCa3.1 channel function reduced cytokine transcription, suggesting that radiation-induced expression is sustained by channel activity. Irradiated human primary GBM cells also increased *IL-4* and *IL-4R* gene expression; however, KCa3.1 inhibition with TRAM-34 (48 h after radiation) only reduced cytokine expression (Figure 4B). We also measured the protein level of IL-4 in GL-15 and in human GBM cells by cytofluorimetry. As shown in Figure 4C, in line with the mRNA expression experiments, radiation increased the level of intracellular IL-4 in GL-15 and GBM18 cells approximately 2-fold (1.89 ± 0.04) and 2.5-fold (2.7 ± 0.1), respectively. KCa3.1 inhibition with TRAM-34 decreased protein expression in both cell cultures (GL-15: 1.3 ± 0.1; GBM18: 2.1 ± 0.2). Intracellular IL-4 was measured in the presence of Brefeldin A (10 µg/mL), which blocks protein transport from the endoplasmic reticulum.

## 3. Discussion

GBM is an invasive brain tumor, able to reach and colonize brain regions far from the original tumoral core. The poor prognosis of GBM patients is primarily associated with the invasive phenotype and the growth rate of GBM cells [23]. We have previously shown that KCa3.1 inhibition reduces GBM invasion in vitro and in mouse models, affecting both glioma cells and microglia [14,21]. We now wanted to verify the hypothesis that KCa3.1 could also be involved in modulating radiation-induced GBM cell invasiveness. In particular, we focused on the effect of radiation doses higher than those used in stereo radio-surgery for recurrent GBM [16]. We demonstrated that this treatment increased the invasive ability of GL-15, a human GBM cell line, and of primary GBM cells derived from patients. GL-15 cells were chosen in this study for their high invasiveness [14,21,24]. Many different reports demonstrated that different radiation protocols stimulated cell migration in different cancers (see [25] for a review) as well as in GBM [26,27,28,29]. However, the mechanisms underlying the induction of a pro-invasive phenotype are still under investigation. Here, we show that cell irradiation with a unique high dose induces gene expression and the functional up-regulation of KCa3.1 channels, as demonstrated by qRT-PCR and electrophysiological recordings in GL-15 cells and in primary GBM cells derived from patients. Recently, in human T98G and U87MG cell lines, fractioned radiation has been shown to up-regulate KCa3.1 channels. In particular, it was reported that the blockade of KCa3.1 radiosensitized these highly proliferative cell lines, impairing their clonogenic properties in vitro and their ectopic growth in vivo [30]. Another calcium-dependent potassium channel, KCa1.1, is up-regulated upon radiation, and its increased activity was associated with the increased invasive behavior of U87MG cells [12]. Here we show that very high doses of radiation also affects GBM cell migration. In all the tested GBM cells, we found an increased ability to invade a Matrigel layer. We also demonstrated that this gained function was related to an increased functional KCa3.1 expression, because channel inhibition with TRAM-34 significantly reduced the radiation-induced cell invasion. Recently, we have shown that KCa3.1 channel function is involved in the activation state of microglial cells and modulates the transcription of inflammatory genes [22]. In the present study, we started from the observation that radiation increased KCa3.1 function, and evaluated the expression of key genes related to cell invasion and/or reported to be increased after radiation in GBM, such as CXCL12 [12,31], CXCR4 [32], EGFR [33,34], and metalloproteinases [35,36]. Our data demonstrate that the altered gene expression profile induced by radiation was sustained by KCa3.1 activity, and was modulated by TRAM-34 treatment of GL-15 cells. In primary GBM cells, radiation boosted gene transcription of several genes among those analyzed, and some key genes were also modulated by KCa3.1 inhibition. We also investigated whether transcription factors involved in the expression of invasion-related genes could be modulated by radiation. Here, we report the increased expression of AP-1, ATF2, REST, and EGR3, all transcription factors involved in cancer cell invasive behavior [36,37,38,39,40]. AP-1 together with ATF2, EGR3, and REST, have top transcription factor-binding sites in the *KCNN4* gene promoter, as evaluated in the QIAGEN database (https://www.genecards.org/cgi-bin/carddisp.pl?gene=KCNN4). The AP-1 dependent transcription of KCa3.1 channels was previously shown in T cells and microglia [17,18]. In contrast, in vascular smooth muscle cells, REST activity suppresses *KCNN4* expression, with effects on cell proliferation [41] likely reflecting cell-specific responses. To our knowledge, this is the first report of EGR3 expression in GBM. We suggest that these results could explain the mechanisms underlying the increased invasive capabilities induced in GBM by very high doses of radiation, such as those occurring upon radiation surgery. One of the pathways involved in *KCNN4* transcription is the signaling through the IL-4/IL-4R pair, together with the activation of AP-1 factor [17,18]. Radiation induces the expression of the *IL-4* gene in human carcinoma cells [42]. In our experiments, we report the increase of *IL-4* and *IL-4R* expression in irradiated GL-15 cells. This is different from other reports on medulloblastoma and GBM cells exposed to a single dose (5 Gy) of gamma radiation, where IL-4R decreased [43]. Furthermore, it must be considered that IL-4, a classical type 2 cytokine, may induce type 1 immunity, increasing survival of glioma-bearing mice and decreasing tumorigenicity of sarcoma cell in vivo [44,45]. Nevertheless, IL-4 also modulates the brain microenvironment in glioma, with effects on tumor activated myeloid cells, and the promotion of tumor growth and invasiveness (reviewed in [46,47]). It has been reported that GBM patients have higher central and peripheral expression of IL-4 [48], and we here describe that the inhibition of the KCa3.1 channel reduces *IL-4* transcription in GL-15 cells, suggesting that radiation-induced expression is sustained by channel activity. Irradiated primary GBM cells showed a similar behavior in terms of *IL-4* and *IL-4R* expression while KCa3.1 inhibition only affects cytokine expression. FACS analysis also supported the increase of intracellular IL-4 in irradiated GL-15 and GBM18 cells, and the key modulatory role of KCa3.1 activity. This finding may have significant implications for future therapeutic approaches, since IL-4 has immune suppressive functions in the GBM microenvironment, acting as inducer of a pro-tumor, anti-inflammatory microglia phenotype [22,47]. To our knowledge, this is the first study describing that GBM cells increase IL-4 expression upon treatment with a high dose of radiation.

To summarize, we propose the following interpretation of our results: in GBM cells, a high dose of radiation induces IL-4 and KCa3.1 expression, with the consequent intracellular calcium mobilization [40] and gene transcription of pro-invasive factors. The small sample size of patient tissues and the high heterogeneity of GBM tumor requires further analyses to obtain conclusive translational data. However, the understanding of these mechanisms of action could have important applications to reduce GBM cell invasiveness in patients, also with the possibility to repurpose Senicapoc^®^, a compound that is structurally similar to TRAM-34 [19] and has been previously tested in clinical trials for other diseases.

## 4. Materials and Methods

### 4.1. Materials

All cell culture reagents D-MEM (high-Glucose), fetal bovine serum (FBS), amphotericin B, penicillin G, streptomycin, and glutamine were from Invitrogen (Carlsbad, CA, USA). All salts and solvents were form Sigma-Aldrich (Saint Louis, MO, USA).

### 4.2. Cell Line Culture and Radiation Protocol

Radiation was performed on sub-confluent GL-15, cultured as previously described [14], and primary GBM cells in T25 flasks in complete growth medium. Cells were exposed to X-ray in a Raycell cabinet system (Ottawa, ON, Canada) calibrated for blood bags with a dose of 35 Gray (Gy) (1 cycle).

### 4.3. Primary Human GBM Cells

Tumor specimens were obtained from the Neurological Science Department of Sapienza Medical School and from Neuromed, from primary GBM in adult patients who gave informed consent to the research proposals (Supplementary Table S2). The study was conducted in accordance with the Declaration of Helsinki, and the protocol was approved by the Ethics Committee of Policlinico Umberto I and Neuromed. Ethic code: 23 April 2015, Rif.3623 Prot.2061/15, Ethics Committee of Policlinico Umberto I; 31 July 2012, Prot. 7/12, Ethics Committee of Neuromed. Tissues were processed within half an hour from surgical resection. Histopathological typing and tumor grading were done according to the WHO criteria, resulting as grade IV. In detail, tumor tissues were mechanically dissociated to cell suspensions and centrifuged at 800× *g* for 5 min. Red blood cells were lysed with 4 vol of ammonium chloride buffer (in mM: 154 NH_4_Cl, 10 K_2_CO_3_, and 0.1 EDTA) at 4 °C for 5 min. Tumor cells were resuspended in serum-free growth medium (D-MEM with 100 IU/mL penicillin G, 100 mg/mL streptomycin, 4 mM glutamine, and 1 mM sodium pyruvate) and cultured at 37 °C in a humidified atmosphere with 5% CO_2_. Twenty-four hours later, non-adherent cells were removed, and the growth medium was supplemented with 10% heat-inactivated FBS, with changes every 48 h. After about 10 days, cells were sub-cultured. In this work we used four primary cultures: GBM18, GBM19, GBM45, and GBM137. These primary cell cultures were chosen because they responded to chemokines in chemotaxis assays and expressed KCa3.1 channels [15,49,50].

### 4.4. Migration and Invasion Assays

Twenty-for hours after irradiation, irradiated or control sub-confluent cells were trypsinized, and plated in invasion medium (DMEM supplemented with 100 IU/mL penicillin G and 100 μg/mL streptomycin, 0.1% BSA and 25 mM HEPES, pH 7.4), at a density of 7 × 10^3^ cells/cm^2^ on poly-lysine or Matrigel-coated transwells (Corning, 8 μm pore size, Corning, NY, USA). Cells were incubated at 37 °C for 4 h (migration) or for 24 h (invasion) with 2 mM hydroxyurea added to both chambers to prevent cell proliferation, then fixed in ice-cold 10% trichloroacetic acid for 10 min in the presence or absence of 5 µM of TRAM-34. Cells adhering to the upper side of the filter were scraped off, whereas cells invaded through the insert were stained with a solution containing 50% isopropanol, 1% formic acid, and 0.5% (w/v) Brilliant blue R 250 (Sigma-Aldrich) and counted on the entire membrane area (at least 20 fields) with a 20× objective.

### 4.5. Electrophysiology

Twenty-for hours after irradiation, GBM cells were plated on petri dishes (2 × 10^4^ cells/dish) and tested after 24–48 h of TRAM-34 treatment (48–72 h after radiation). The whole-cell, patch-clamp configuration was used for electrophysiological recordings. Currents were amplified with a HEKA EPC-10 amplifier (List Medical, Darmstadt, Germany), digitized with a 12-bit A/D converter (TL-1, DMA interface; Axon Instruments, Foster City, CA, USA), and analyzed with the Patch Master package (version 2 × 60, Heka Elektronik, Holliston, MA, USA) and Microcal Origin 6.0 software. For on-line data collection, macroscopic currents were filtered at 3 kHz and sampled at 50 µs/point. The external solution contained (in mM): NaCl 140, KCl 5, CaCl_2_ 2, MgCl_2_ 2, MOPS 5, glucose 10, (pH 7.4). The internal solution contained (in mM): KCl 150, EGTA-K 1, MgCl_2_ 1, MOPS 5, Na_2_ATP 5 (pH 7.20). CaCl_2_ was added to the internal solution to have a free Ca^2+^ concentration of 1 µM as assessed using the program Webmax (https://web.stanford.edu/~cpatton/webmaxcS.htm). Access resistances ranging between 5 and 15 MΩ were achieved and actively compensated to 50%. Following the establishment of the whole-cell configuration, cells were stimulated by applying 1 s voltage ramps from −90 to 20 mV from a holding potential of −60 mV. In order to achieve complete exchange of the cytoplasm solution, electrophysiological experiments began 3 min after the achievement of the whole cell configuration. After this time, the current ramps were sufficiently stable over time. The KCa3.1 channel agonist SKA-31 (3 µM, Sigma-Aldrich) or NS309 (10 µM, Sigma-Aldrich) was applied to maximally activate the KCa3.1 current, and then 3 µM TRAM-34 was added, together with the channel agonist, to estimate the maximal KCa3.1 current. The current density was assessed by dividing the current recorded at 0 mV by the cell capacitance measured by the amplifier. Data were presented as mean +/− standard error. The one-way ANOVA test was applied to assess significant differences among the three experimental groups (Control, Irradiated, and Irradiated plus TRAM-34, Supplementary Figure 1), and the Student’s *t*-test was applied to detect differences between groups. In both cases, a *p* < 0.05 was considered to be significant.

### 4.6. RNA Preparation and Analysis

Total RNA was isolated from cell cultures (irradiated or not) treated for 48 h with TRAM-34 or vehicle. Treatments started 24 h after irradiation. RNA was isolated using Trizol reagent (Ambion, Life Technologies, Carlsbad, CA, USA) according to the manufacturer’s instructions. The cDNA was prepared using the iScript Reverse Transcription Supermix (Bio-Rad Laboratories, Hercules, CA, USA); quantitative real time PCR was performed using the SsoFast Evagreen Supermix (Bio-Rad Laboratories) according to the protocol for use in the Biorad I cycler System. For the quantification analysis, the comparative threshold cycle (Ct) method was used. The Ct values of each gene were normalized to the Ct value of GAPDH in the same RNA sample. The gene expression levels were evaluated by fold changes using the equation 2^-ddCt^. The primers used are reported in Table 1.

### 4.7. Flow Cytometry Analysis

For evaluation of IL-4 production, GBM cells were treated with TRAM-34 (5 µM for 48 h) 24 h after irradiation, maintained in complete medium in the presence of Brefeldin A (10 µg/mL) for the last 4 h, and then detached with trypsin/EDTA. Cells were labeled with the fixable viability dye eFluor^TM^ 780 (Invitrogen) to exclude dead cells from the analysis and stained using the BD Cytofix/Cytoperm^TM^ fixation/permeabilization kit according to the manufacturer’s instructions. APC mouse anti-human IL-4 Ab (clone 8D4-8) was from BD Pharmingen and staining was performed at 4 °C for 20 min; the corresponding Ab isotype was used as negative control. Samples were analyzed using a flow cytometer FACS-Canto II (BD Biosciences) and data were elaborated using FlowJo 9.3.2 software (BD Biosciences, San Jose, CA, USA). Quantification of IL-4 expression was reported as the fold-increase of median fluorescence intensity of each sample versus control minus isotype value.

### 4.8. Statistical Analyses

Data were expressed as the mean ± SEM. Statistical significance was assessed by Student’s *t*-test or one-way ANOVA followed by Student–Newman–Keuls method of comparison. All statistical analyses were done using Sigma Plot 11.0 software (https://systatsoftware.com).

## 5. Conclusions

Data reported in this manuscript demonstrate that GBM cells irradiated with a single high dose of 35 Gy to mimic the condition of stereotaxic radio-surgery performed in patients, increased the invasion ability, by increasing the transcription level of genes involved in invasion and other in transcription regulation. We found that the activity of KCa3.1 channels, whose functional expression increased in irradiated cells, modulates these effects. We also found that a high radiation dose stimulates the transcription of IL-4 and IL-4 receptors in GBM cells, again counteracted by KCa3.1 inhibition. This result suggests that high radiation can promote a more aggressive tumor phenotype, also because IL-4 acts as an immunosuppressant, regulating GBM microenvironment. These effects can be modulated by the activity of KCa3.1.

## Figures and Tables

**Table 1:** Primers used in RNA analysis.

AP-1	Forward: 5′-AGAGGAAGCGCATGAGGAAC-3′ Reverse: 5′-CACCTGTTCCCTGAGCATGT-3′
ATF2	Forward: 5′-GGCCAATTGTCCCTGTACCA-3′ Reverse: 5′-ACCATGGTGACTGGTCGAAC-3′
CXCL12	Forward: 5′-AGCCAACGTCAAGCATCTCA-3′ Reverse: 5′-GTGGGTCTAGCGGAAAGTCC-3′
CXCR4	Forward: 5′-CCCTCCTGCTGACTATTCCC-3′ Reverse: 5′-TAAGGCCAACCATGATGTG-3′
EGR3	Forward: 5′-AGTTTGCTAAACCAACTGCC-3′ Reverse: 5′-TTGGTCAGACCGATGTCCAT-3′
GAPDH	Forward: 5′-CCCCTTCATTGACCTCAACTAC-3′ Reverse: 5′-GATGACAAGCTTCCCGTTCTC-3′
IL-4	Forward: 5′-GACTCTGTGCACCGAGTTGA-3′ Reverse: 5′-TCAGGAATCGGATCAGCTGC-3′
IL-4R	Forward: 5′-ATGAAGGTCTTGCAGGAGCC-3′ Reverse: 5′-ACGTCATCCATGAGCAGGTG-3′
KCNN4	Forward: 5′-GGCTGAAACACCGGAAGCTC-3′ Reverse: 5′-CAGCTCTGTCAGGGCATCCA-3′
MMP2	Forward: 5′-AGATCTTCTTCTTCAAGGACCGGTT-3′ Reverse: 5′-ACAGCCTTCTCCTCCTGTGG-3′
MMP9	Forward: 5′-AACTACGACCGGGACAAG-3′ Reverse: 5′-TCCGCGGCCCTCGCTGGTAC-3′
MMP12	Forward: 5′-AGGAATCGGGCCTAAAATTG-3′ Reverse: 5′-TGCTTTTCAGTGTTTTGGTGA-3′
REST	Forward: 5′-GTTAGAACTCATACAGGAGAA-3′ Reverse: 5′-AACTGCACTGATCACATTTAA-3′

## Supplementary Materials

The following are available online at https://www.mdpi.com/2072-6694/11/3/279/s1, Figure S1: MTT assay on irradiated GL-15 cells, Figure S2: Radiation-induced KCa3.1 activity is repressed after TRAM-34 treatment, Figure S3: Survival analysis based on expression of the indicated genes, Table S1: The effects of radiation (IRR) and radiation + TRAM-34 (IRR+TRAM-34) treatment on gene expression in each cell population, Table S2: GBM patient details.
