# Supplementary material for: Radiation Increases Functional KCa3.1 Expression and Invasiveness in Glioblastoma
Source: Cancers (Basel). 2019 Feb 26;11(3):279. doi: 10.3390/cancers11030279 (PMC6468446; doi:10.3390/cancers11030279)
Supplement: Supplementary file 1 [file cancers-11-00279-s001.pdf]

Supplementary Materials: Radiation Increases Functional KCa3.1 Expression and Invasiveness in Glioblastoma

Giuseppina D'Alessandro, Lucia Monaco, Luigi Catacuzzeno, Fabrizio Antonangeli, Antonio Santoro, Vincenzo Esposito, Fabio Franciolini, Heike Wulff and Cristina Limatola

Supplementary Method

MTT Assay

Twenty-four hours after irradiation, GL-15 cells (10^4/well) were seeded in 96 well-plate and assayed for their viability after further 48 hours. After this time, MTT salt (500 µg/mL) was added into each well for 1.5 h. DMSO was then added to stop the reaction and the formazan produced was measured at 570 nm. Viability of cells was expressed relative to absorbance.

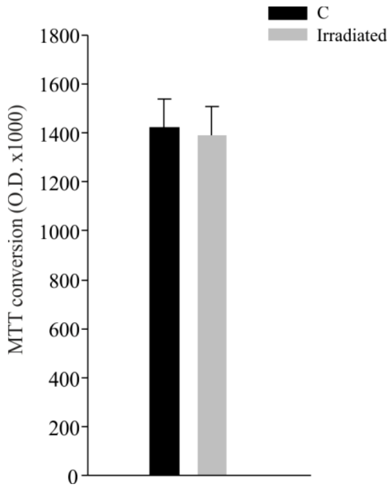

Figure S1. MTT assay on irradiated GL-15 cells. Cell viability did not show significantly difference between GL-15 control (C) and GL-15 Irradiated 72 h after irradiation protocol; n = 3 (six replicate each) by Student *t*-test.

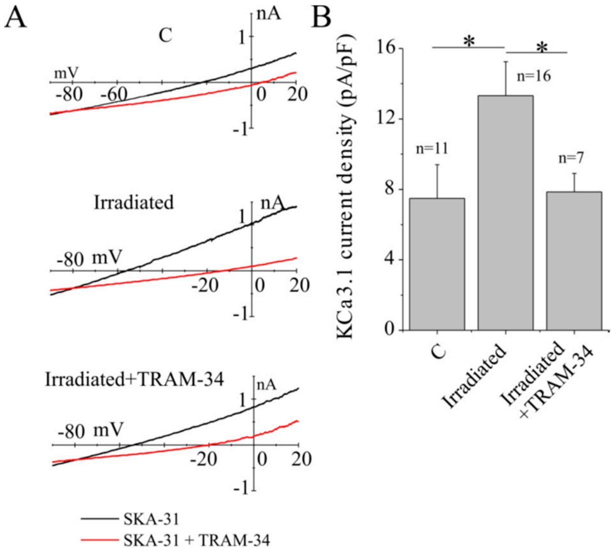

Figure S2. KCa3.1 current in irradiated GL-15 cells. (**A**) Current voltage relationships obtained by applying voltage ramps from −90 to +20 mV, from a holding potential of −60 mV, to GL15 cells

cultured at the indicated three different conditions: control (C), Irradiated and Irradiated+TRAM-34 for 48h. Black traces are in presence of external SKA-31 (3 μM), while red traces are in presence of external SKA-31 (3 μM) + TRAM-34 (3 μM). Irradiated +TRAM-34 cells were recorded after a minimum time of 30 min washing in the external recording solution, in order to allow revertion of the inhibitory effect of TRAM-34. External recording solution (in mM): NaCl 140, KCl 5, $CaCl_2$ 2, $MgCl_2$ 2, MOPS 5, glucose 10, pH 7.4 with NaOH. Internal recording solution: KCl 155, EGTA-K 1, $MgCl_2$ 1, MOPS 5, pH 7.2 with KOH. $CaCl_2$ was added to the internal solution to obtain a free Ca^{2+} concentration of 1 μM. (**B**) Mean KCa3.1 current densities (current to electrical capacitance ratio) assessed at 0 mV as the TRAM-34 sensitive current. Data were obtaining using two different KCa3.1 channel activators (NS309 10 μM, *n* = 7, 13, and 6 for C, Irradiated, and Irradiated+TRAM-34 cells; SKA-31 3 μM, *n* = 4, 3, and 1 for the C, Irradiated, and Irradiated+TRAM-34 cells) were pooled together. * $p < 0.05$ by One-Way ANOVA.

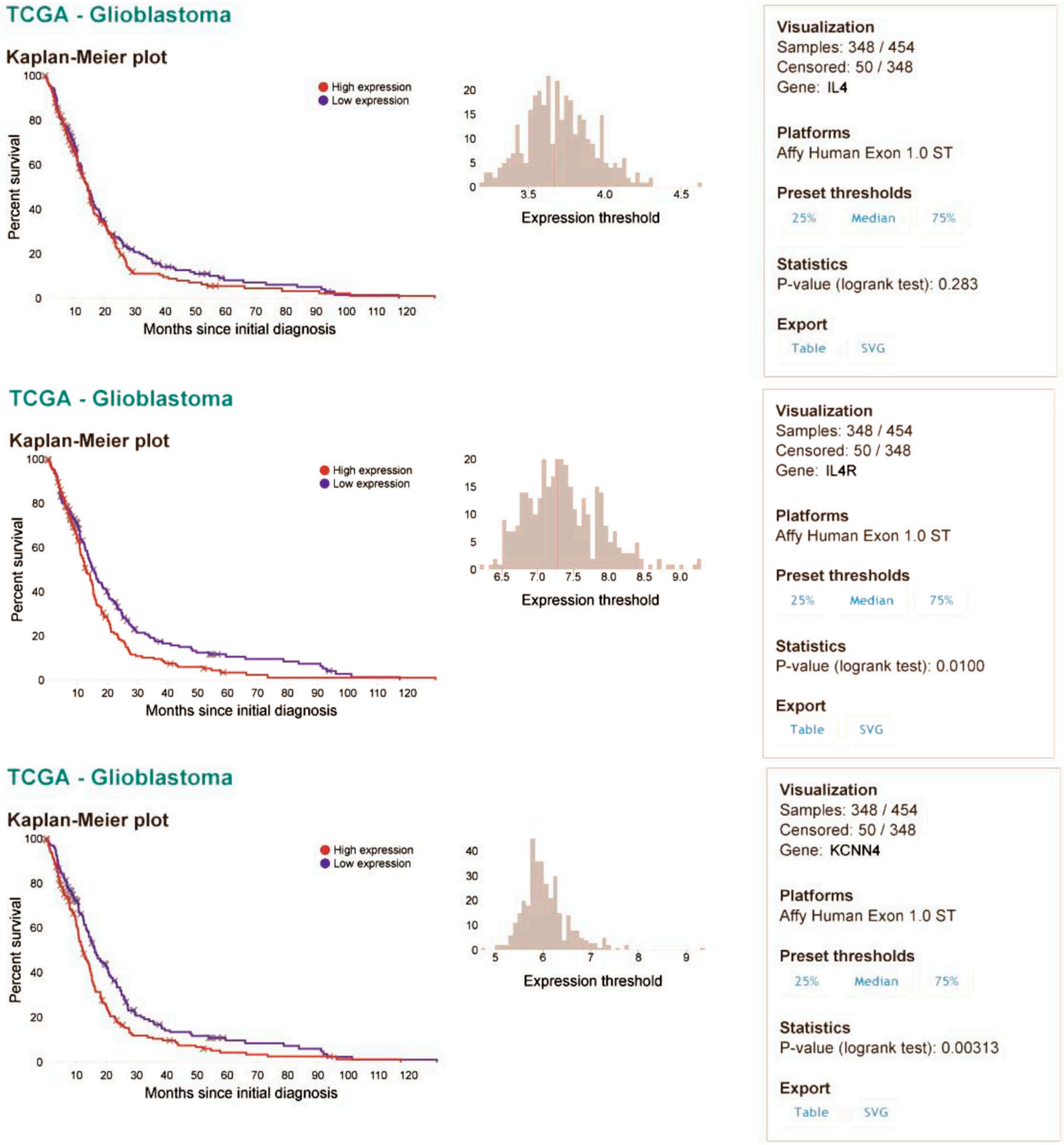

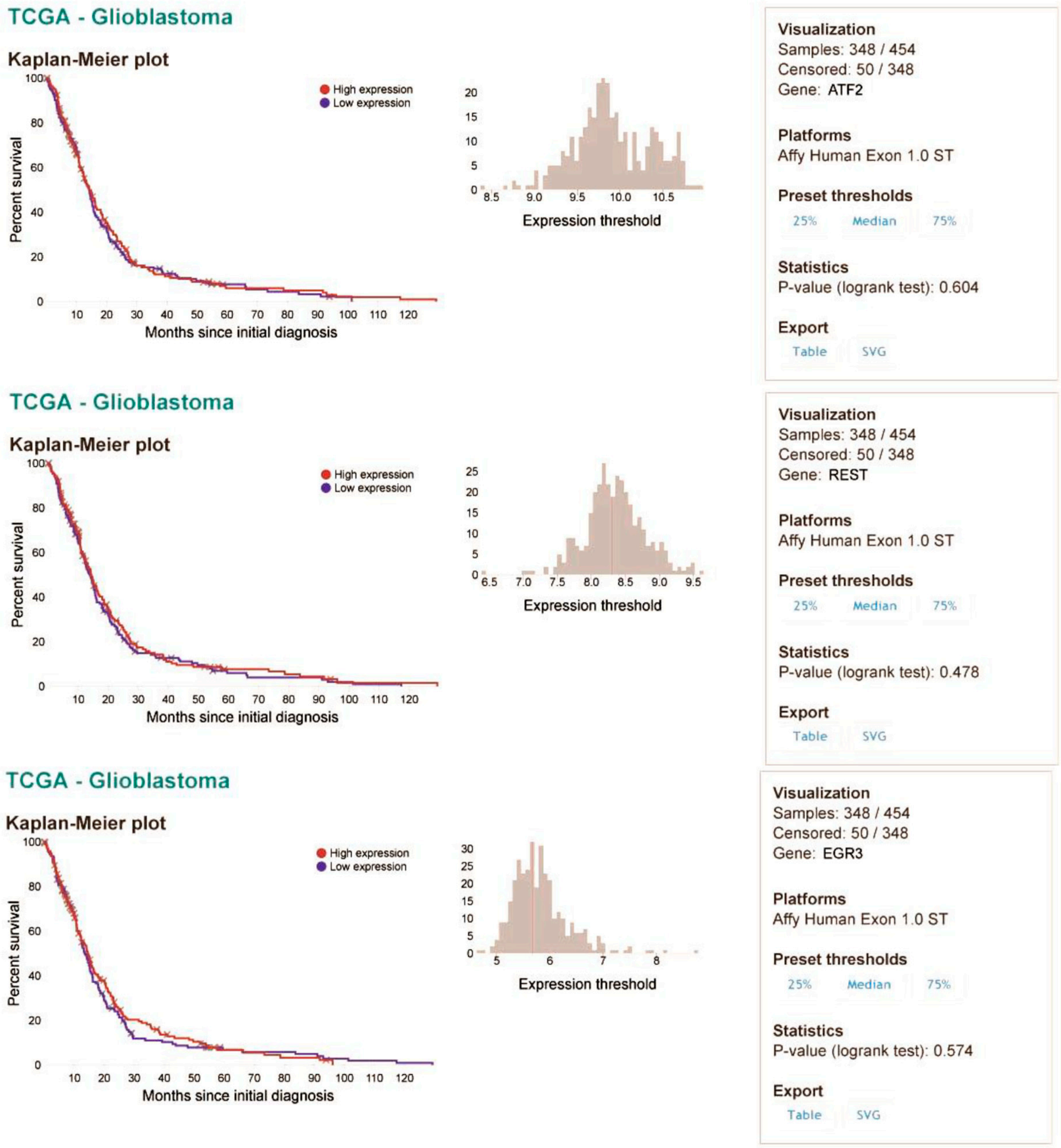

Figure S3. Survival Analysis based on gene expression. Survival analysis based on expression of the indicated genes. Analysis was performed using the Affymetrix Human Exon 1.0 ST platform (TCGA GBM dataset, project Betasasis).

Table S1. The effects of radiation (IRR) and radiation + TRAM-34 (IRR+TRAM-34) treatment on gene expression in each cell population. Values are shown as mRNA fold increase over respective not irradiated cells of indicated genes. Each primary cell culture was tested twice.

Gene	GBM18		GBM19		GBM45		GBM18		GBM19		GBM45	
	IRR		IRR		IRR		IRR + TRAM		IRR + TRAM		IRR + TRAM	
**AP1**	**1.1**	**1.1**	**1.3**	**1.2**	**1.8**	1.8	0.9	0.9	2.2	2.2	1.1	1.1
**ATF2**	**1.3**	**1.4**	**1.8**	**1.9**	**1.9**	**2.0**	**0.5**	**0.5**	**1.8**	**1.7**	**0.7**	**0.7**
**EGR3**	12.7	12.3	6.6	6.6	4.4	4.4	6.8	6.5	2.6	2.6	2.3	2.2
**REST**	**0.9**	**1.0**	**0.9**	**0.9**	**1.5**	**1.5**	**0.6**	**0.6**	**1.3**	**1.3**	**0.8**	**0.8**
**CXCL12**	7.2	7.0	3.5	3.7	2.2	2.4	2.7	3.0	3.0	3.1	3.3	3.3
**CXCR4**	**4.4**	**4.4**	**3.5**	**3.5**	**2.3**	**2.4**	**1.6**	**1.6**	**3.3**	**3.3**	**0.8**	**0.8**
**MMP2**	6.9	6.7	4.6	4.6	1.3	1.2	3.6	3.5	8.5	8.0	1.0	1.1
**MMP9**	17.6	18.0	3.2	3.3	3.0	3.0	3.9	3.9	0.5	0.6	0.5	0.5
**KCNN4**	20.1	21.0	10.9	10.4	5.8	6.0	7.5	7.5	0.5	0.5	2.6	2.8

Table S2. GBM patient's information.

Name.	Age (Years)	Sex	Grade (WHO)	Relapse (Y/N)
GBM18	73	Male	IV	N
GBM19	79	Male	IV	N
GBM45	62	Female	IV	N
GBM137	72	Male	IV	N

© 2019 by the authors. Licensee MDPI, Basel, Switzerland. This article is an open access article distributed under the terms and conditions of the Creative Commons Attribution (CC BY) license (http://creativecommons.org/licenses/by/4.0/).
